# Memory CD4+ T-Cell Lymphocytic Angiopathy in Fatal Forms of COVID-19 Pulmonary Infection

**DOI:** 10.3389/fimmu.2022.844727

**Published:** 2022-04-22

**Authors:** Amélie Guihot, Isabelle Plu, Cathia Soulié, Alice Rousseau, Cecilia Nakid-Cordero, Karim Dorgham, Christophe Parizot, Elena Litvinova, Julien Mayaux, Isabelle Malet, Paul Quentric, Béhazine Combadière, Christophe Combadière, Olivia Bonduelle, Lucille Adam, Pierre Rosenbaum, Alexandra Beurton, Patrice Hémon, Patrice Debré, Vincent Vieillard, Brigitte Autran, Danielle Seilhean, Frédéric Charlotte, Anne-Geneviève Marcelin, Guy Gorochov, Charles-Edouard Luyt

**Affiliations:** ^1^Sorbonne Université INSERM, Centre d’Immunologie et des Maladies Infectieuses (CIMI-Paris), Hôpital Pitié-Salpêtrière, Paris, France; ^2^Assistance Publique-Hôpitaux de Paris (AP-HP), Hôpital Pitié-Salpêtrière, Département d’Immunologie, Paris, France; ^3^Sorbonne Université, Assistance Publique-Hôpitaux de Paris (AP-HP), Hôpital Pitié-Salpêtrière, Département de Neuropathologie, Paris, France; ^4^Sorbonne Université, INSERM, Institut Pierre Louis d’Epidémiologie et de Santé Publique (iPLESP), AP-HP, Hôpital Pitié Salpêtrière, Laboratoire de Virologie, Paris, France; ^5^Assistance Publique-Hôpitaux de Paris (AP-HP), Hôpital Pitié-Salpêtrière, Service de Médecine Intensive–Réanimation et Pneumologie, Paris, France; ^6^LBAI, Hyperion platform, University of Brest, INSERM, CHU de Brest, Brest, France; ^7^Assistance Publique-Hôpitaux de Paris (AP-HP), Service d’Anatomopathologie, Hôpital Pitié-Salpêtrière, Paris, France; Sorbonne Université, Paris, France; ^8^Assistance Publique–Hôpitaux de Paris (AP-HP), Hôpital Pitié–Salpêtrière, Service de Médecine Intensive Réanimation, Institut de Cardiologie, Paris, France; ^9^Sorbonne Université, Inserm, Institute of Cardiometabolism and Nutrition (ICAN), Paris, France

**Keywords:** COVID-19, T cell responses, Broncho-alveolar lavage (BAL), Autopsia, Vasculitis

## Abstract

The immunopathological pulmonary mechanisms leading to Coronavirus Disease (COVID-19)-related death in adults remain poorly understood. Bronchoalveolar lavage (BAL) and peripheral blood sampling were performed in 74 steroid and non-steroid-treated intensive care unit (ICU) patients (23–75 years; 44 survivors). Peripheral effector SARS-CoV-2-specific T cells were detected in 34/58 cases, mainly directed against the S1 portion of the spike protein. The BAL lymphocytosis consisted of T cells, while the mean CD4/CD8 ratio was 1.80 in non-steroid- treated patients and 1.14 in steroid-treated patients. Moreover, strong BAL SARS-CoV-2 specific T-cell responses were detected in 4/4 surviving and 3/3 non-surviving patients. Serum IFN-γ and IL-6 levels were decreased in steroid-treated patients when compared to non-steroid treated patients. In the lung samples from 3 (1 non-ICU and 2 ICU) additional deceased cases, a lymphocytic memory CD4 T-cell angiopathy colocalizing with SARS-CoV-2 was also observed. Taken together, these data show that disease severity occurs despite strong antiviral CD4 T cell-specific responses migrating to the lung, which could suggest a pathogenic role for perivascular memory CD4 T cells upon fatal COVID-19 pneumonia.

## Introduction

Severe COVID-19 infection mainly occurs in the elderly or in patients with vascular comorbidities including hypertension or diabetes (1). The SARS-CoV-2 virus infects type I and type II lung pneumocytes and possibly also endothelial cells that harbor the SARS-CoV-2 receptor ACE-2 (2, 3). In more severe forms of COVID-19 infection, the SARS-CoV-2 nasopharyngeal viral load is high, and the virus can also be found in peripheral blood (4). This viremia is frequent in fatal forms of infection (5).

We and others have shown that at the acute phase, the biological profile is dominated by an inflammatory response involving cytokines such as IL-6, TNF-α and IFN-γ induced chemokines such as IP-10 (6–8). Concomitantly, a global lymphopenia during the most severe forms of the disease is observed (9). Whether the lymphopenia observed reflects the pulmonary trafficking of immune cells to the lungs or to the lymph nodes, *via* cytokines such as IFN-I that may promote T-cell attachment to endothelium (10), is still unknown. Moreover, the immune mechanisms underlying the partial efficacy of steroids is poorly defined (11).

During the more severe forms of the disease, a strong humoral and cellular immune response to the virus is observed. Patients achieve an IgA and IgG seroconversion within 20 days after symptoms appear (12). A robust activated CD4 and CD8 T-cell response has been associated with clinical severity, while mortality could not be linked with a distinct immunophenotype (13, 14). Thus, for very severe COVID-19 infection, the immune mechanisms leading to death are still largely unknown, particularly whether the local immune response is protective or if, on the contrary, it leads to immunopathology mechanisms resulting in lung injury, as observed during fatal forms of pulmonary influenza infection, which has been linked to pulmonary immune complex deposition (15), the trapped antibodies being influenza specific (16).

Here, we characterized the systemic and pulmonary local immune response in an intensive care unit (ICU) in adults with favorable or fatal COVID-19 infection. We found evidence for a Th1 CD4 polarization of pulmonary T lymphocytes, with a high frequency of SARS-CoV-2-specific T cells at the alveolar level. Furthermore, we show a memory CD4 T-cell vasculitis in lungs from 3 additional autopsies.

## Methods

### Patients

All consecutive first-wave patients admitted in two ICUs in the Pitié-Salpêtrière Hospital between 03/18/20 and 04/24/20 (first wave) and between 10/02/20 and 11/09/20 (second wave) with laboratory-confirmed SARS-CoV-2 infection, documented by real-time reverse trascription polymerase chain reaction (RT-PCR) on nasopharyngeal swabs, or a lower respiratory specimen [tracheal aspirate or bronchoalveolar lavage (BAL)], and who required mechanical ventilation were included. In accordance with the current French law, informed written consent was obtained from patients and/or relatives. The protocol was approved by our institution’s ethics committee (Immuno-COVID-REA, CER-Sorbonne Université, no. CER-SU-2020-31). Acute respiratory distress syndrome (ARDS) was defined according to the Berlin definition (17). The most severe patients were put on veno-venous extracorporeal membrane oxygenation (ECMO), according to predefined criteria (17). Briefly, the patients eligible for ECMO had to fulfill the ARDS criteria, and one of the following disease severity criteria, despite ventilator optimization (fraction of inspired oxygen [FiO_2_] ≥80%, tidal volume set at 6 ml/kg predicted bodyweight, and positive end-expiratory pressure ≥10 cm of water): (1) partial pressure of arterial oxygen (PaO_2_) over an FiO_2_ ratio of less than 50 mm Hg for more than 3 h; (2) PaO_2_/FiO_2_ less than 80 mm Hg for more than 6 h; or (3) arterial blood pH less than 7·25 with a partial pressure of arterial carbon dioxide (PaCO_2_) of 60 mm Hg or more for 6 h or more (18). The proportion of ECMO patients was higher during the second wave than during the first wave in our center, due to the special recruitment and expertise of the ICU (**Tables 1**, **2**).

**Table 1:** Very severe ICU patients with COVID-19 infection: first wave.

Patient#	Age	Sex	First symptoms to first sample delay	Pre-existing conditions	SAPS II score on ICU admission	SOFA score on ICU admission	ARDS	ECMO	Outcome: 0 deceased; 1 survivor
1	58	M	6	Obesity, HBP	70	19	1	1	0
2	68	M	8	Diabetes, obesity, HBP	38	14	1	0	0
3	49	M	8	Diabetes	24	5	1	0	1
4	75	M	7	Immunosupression	49	9	1	0	0
5	75	M	12	Diabetes, obesity	45	10	1	0	1
6	60	F	13	Diabetes, obesity, HBP	50	16	1	1	1
7	54	M	23	Obesity	26	7	1	1	1
8	69	M	8	Diabetes, obesity, HBP, immunosuppression	46	10	1	0	0
9	34	F	8	Diabetes, obesity, HBP	52	20	1	1	0
10	56	F	17	Obesity	54	12	1	1	0
11	48	M	23	Diabetes, obesity	46	11	1	1	1
12	64	M	9	Obesity	37	6	1	0	0
13	71	F	6	Obesity, HBP	54	7	1	0	0
14	63	F	12	HBP, asthma	65	16	1	1	1
15	58	M	14	Immunosupression	37	7	1	0	0
16	50	M	6	Infarction, obesity	26	10	1	1	1
17	51	M	22	HBP, immunosuppression	66	13	1	1	0
18	58	M	14	HBP	39	16	1	1	1
19	53	M	13	None	27	4	1	1	1
20	36	M	NA	Respiratory disease	33	7	1	0	1
21	69	M	10	Cardiac condition	14	3	1	0	0
22	54	F	5	Respiratory disease, obesity	25	8	1	1	1
23	42	M	11	Diabetes, obesity, HBP	54	12	1	1	1
24	55	M	8	Obesity	32	17	1	1	1
25	49	M	8	Obesity, HBP	61	14	1	1	1
26	61	M	17	Obesity, HBP	38	5	1	0	1
27	51	M	12	Diabetes, HBP	44	17	1	1	1
28	65	M	14	Diabetes, HBP	50	9	1	1	1
29	79	F	5	None	37	3	0	0	0
30	64	M	6	HBP, obstructive pulmonary disease, diabetes, obesity	24	7	1	0	0
31	72	M	16	Obesity, respiratory disease, cardiac condition	36	4	1	0	0
32	23	M	12	Respiratory disease, diabetes, obesity	56	12	1	1	1
33	55	M	8	Cardiac condition, immunosuppression	40	6	1	0	0
34	60	M	7	Cardiac condition	27	2	1	0	1
35	52	M	11	None	29	7	1	1	1
36	25	M	5	None	47	14	1	1	1
37	25	M	7	Obesity	34	4	1	1	1
38	54	M	12	Diabetes	78	14	1	1	1
39	40	M	9	None	52	12	1	1	1
40	36	M	12	None	52	12	1	1	1
41	59	M	2	HBP, obesity	70	19	1	1	1
42	32	M	6	None	24	4	1	0	1
43	54	F	11	Respiratory disease	41	11	1	1	1
44	58	M	10	HBP, asthma, diabetes	44	9	1	0	0
45	71	M	5	Cardiac condition, diabetes	35	7	1	0	0
46	48	M	11	Diabetes, obesity	42	20	1	1	0
Median	55		10		42	10			
IQR	13		5		14	5			

HBP, high blood pressure; ARDS, acute respiratory distress syndrome; ECMO, extracorporeal membrane oxygenation; SAPS, simplified acute physiology score; SOFA, sequential organ failure assesment.

**Table 2:** Very severe ICU patients with COVID-19 infection: second wave.

Patient#	Age	Sex	First symptoms to first sample delay	Pre-existing conditions	Steroid treatment delay	SAPS II score on ICU admission	SOFA score on ICU admission	ARDS	ECMO	Outcome: 0 deceased; 1 survivor
1	54	F	13	Diabetes, obesity, HBP	8	73	15	1	1	0
2	58	H	19	HBP	7	60	8	1	1	1
4	47	F	13		3	70	17	1	1	1
5	62	H	17	Asthma	9	63	11	1	1	1
6	62	H	13	Obesity, HBP	13	69	10	1	1	0
7	54	H	28		20	73	11	1	1	0
8	68	F	22	Diabetes	16	68	10	1	1	0
9	56	H	28	HBP	11	61	5	1	1	1
10	64	F	17	HBP	16	91	18	1	1	0
11	33	F	10	Diabetes, obesity, post-partum	4	27	11	1	1	1
12	61	H	24	HBP	11	82	19	1	1	1
13	59	H	20	Diabetes, obesity, HBP	10	65	8	1	1	0
14	52	F	19	Diabetes, obesity, HBP	14	64	13	1	1	1
15	50	H	22		17	55	14	1	1	1
16	64	F	18	Diabetes, obesity, HBP	12	71	13	1	1	0
17	70	F	8	Diabetes, obesity, renal failure	long-term TT	57	15	1	0	1
18	64	H	25	Obesity	15	68	8	1	1	1
19	43	F	12	Renal transplant	long-term TT	58	13	1	1	0
20	46	H	22	Renal transplant	long-term TT	64	9	1	1	0
21	45	H	10	Diabetes, obesity	9	39	14	1	1	1
22	57	H	4	HBP	1	35	8	1	1	1
23	29	H	22		13	66	15	1	1	NA
24	34	H	16	Obesity	8	56	8	1	1	1
25	62	H	6	Diabetes, obesity, HBP	4	46	15	1	1	1
26	56	H	22	Diabetes, HBP	14	53	13	1	1	0
27	36	H	10		6	51	8	1	1	1
28	53	H	17	Diabetes, obesity, HBP	11	73	12	1	1	1
Median	56		17		11	64	12			

At the time of the study, the standard of care in the two participating ICUs was supportive treatment (i.e., mechanical ventilation, antibiotics in case of bacterial infection, and organ support). Hydroxychloroquine with or without azithromycine, remdesivir, corticosteroids, and anti-IL6 antibodies were not part of the standard of care for the first wave. Steroid treatment was part of the standard care for the second-wave patients. None of the patients received anti-inflammatory treatment during their ICU stay. When ventilator-associated pneumonia was suspected, the patients underwent fiberoptic bronchoscopy and BAL for distal respiratory secretion sampling (19, 20). The BAL fluid was sent to the microbiology department for bacterial cultures and to the immunology department. Autopsy sections were performed in one non-ICU and two ICU cases and were not part of the original study (see *Autopsies* section).

#### SARS-CoV-2 Serology

The detection of IgG or IgA isotype antibodies against the S1 domain of the spike (S) protein was measured and interpreted using commercially available enzyme-linked immunosorbent assay (ELISA) according to manufacturer’s instructions (SARS-CoV-2 IgG and IgA ELISAs; EuroImmun Bussy St Martin, France). For the dissociation of antibody–antigen immune complexes, the serum was treated with a dissociation buffer (1.5 M glycine [pH: 2.8]) and immune complexes were dissociated for 1 h at 37°C. The reaction was stopped by adding a neutralization buffer (1.5 M Tris-HCL [pH: 9.7]).

ELISpot IFN-γ assays were performed as previously described (21) using Diaclone’s ELISpot IFN-γ-pair-antibodies; briefly, 10^5^ peripheral blood mononuclear cells (PBMCs)/well were plated (Merck Millipore, Molsheim, France) in triplicates with the medium, phytohemagglutinin (2 µg/ml; Sigma-Aldrich Saint Louis, Missouri, USA), or SARS-CoV-2-peptide pools (2 µg/ml). The SARS CoV-2 overlapping 18-mer-peptide pools were tested separately: 4 pools for the nucleocapsid (15 peptides each) and 2 pools for the spike protein (158 peptides each). Plates were developed with a streptavidin–alkaline phosphatase conjugate (Amersham, Freiburg, Germany) and an nitroblue tetrazolium chloride 5-brom-4chloro-3'-indolyphosate p-toluidine salt (NBT/BCIP) substrate (Sigma-Aldrich) then air-dried for 24 h before spot-forming cell (SFC) units were read (AID Elispot reader; Autoimmun Diagnostika GmbH, Straßberg, Germany). Results are expressed as mean SFC × 10^6^ from triplicates after background subtraction. The positivity threshold was set at 50 SFC/10^6^ PBMC.

BAL lymphocyte phenotyping was performed after filtration, two wash procedures of BAL cells, and staining with tetraCHROME CD45-FITC/CD4-PE/CD8-ECD/CD3-PC5 Antibody Cocktail (Beckman Coulter Brea, CA, USA). Data were acquired and analyzed on a Navios flow cytometer (Beckman Coulter).

#### Cytokine Dosage

Whole blood and BAL were collected in tubes without an anticoagulant. Sera and BAL supernatants were separated by centrifugation and stored at -80°C less than 2 h after sampling. IL-17A, IL-18, granulocyte macrophage colony stimulating factor (GM-CSF), and IFN-α concentrations were measured using a Simoa (single-molecule array) HD-1 Analyzer™ (Quanterix, Lexington, MA, United States). IL-1β, IFN-γ, IL-6, IL-8, IL-22, TNF-α, and IL-10 concentrations were determined using a multiplexed assay on the Quanterix SP-X™ statistical analysis. Serum IFN-β levels were quantified using a highly sensitive ELISA kit (PBL Assay Science, Piscataway, NJ, United States) based on a two-step assay, according to the manufacturer’s instructions. The concentration of each cytokine in unknown samples was interpolated from the calibration curve by multiplying by the dilution factor. Samples with non-detectable values were replaced by the limit of detection (LOD) value, while those over the detection range were replaced by the upper limit of quantification (ULOQ) (see details in Dorgham et al., JACI, 2021). There were serum cytokine dosages under the LOD for IL-1β, IFNγ, IFNα and IFNβ. In BAL, IFNγ, IL-6, IL-22, IL-10, IL-17, GM-CSF, IFNα", and IFNβ dosages were under the LOD. The cytokine dosage with the same techniques in 11 healthy controls are reported in **Supplementary Table 1**. The Mann–Whitney U test was used when appropriate using Prism 5 (Graph Pad, La Jolla, CA, United States) software. Unsupervised principal component analysis was performed using R 3.6.2 with the Factoextra and FactoMiner functions, using the log-transformed cytokine concentrations of the patients in the serum and BAL.

#### Autopsies

The autopsies of three patients who died from COVID-19 were performed in the neuropathology department of Pitié Salpêtrière Hospital in Paris, France in compliance with the legal rules in force, after the computerized national refusal register (RNR) had been checked and after the relatives had asserted that the patient was not explicitly opposed to a postmortem. The relatives were informed of the use of samples for research. The autopsy cases were sampled in accordance with the COVITIS biobank protocol, approved by the French biomedicine agency (Agence de la Biomédecine, PFS 20-008; French ministry of research DC2020-4022). Lungs were sampled for histopathological analysis. They were fixed for 5 days in 4% formaldehyde before embedding in paraffin, cutting to 3 microns thick, and staining. Lung frozen samples were also taken for performing a SARS-CoV-2 RT-PCR (PATHOCoV study, Sorbonne University). The slides were stained with hematoxylin and eosin (H&E), Masson’s trichrome, and periodic acid Schiff (PAS). The immunostaining was performed by an automate (Ventana BenchMark Stainer; Roche Bâle, Switzerland). The biotinylated secondary antibody was included in the detection kit (Ventana Medical Systems Basic DAB Detection Kit 250-001). The streptavidin–biotin-peroxidase complex was revealed by diaminobenzidine. Antibodies against the following antigens were used: CD3 (Ventana; 2GV6; prediluted), CD20 (Agilent; L26; 1:100), and SARS COV–spike (Abcam; ab 272420; 1:100). A control case who died from an H1N1 influenza in 2009 was used for the comparison of the patterns of immunohistochemistry.

#### Tissue Staining and Imaging Mass Cytometry Image Acquisition

Two successive formalin-fixed paraffin embedded (FFPE) sections of 5 μm were used. The first section was stained with hematoxylin–eosin–saffron (HES) to allow the anatomopathologist to select the regions of interest (ROIs). The second section was stained with the imaging mass cytometry (IMC) South San Francisco, CA, USA panel containing the 30 metal-conjugated antibodies and cell intercalator. Sections from the lung, lymph node, and spleen were cut onto glass slides. The sections were de-paraffinized with xylene and carried through sequential rehydration from 100% ethanol to 70% ethanol before being transferred to TBS. Heat-induced antigen retrieval was performed in a water bath at 95°C for 20 min in a Tris/ethylene diamine tetra acetic (EDTA) buffer (10 mM Tris, 1 mM EDTA, pH9). Slides were cooled to room temperature (RT) and were subsequently blocked with TBS+3%BSA for 30 min at RT. Each slide was incubated with 100 μl of the antibody cocktail (**Supplementary Table 3**) overnight at 4°C. Then, slides were washed 3 times with TBS and labeled with a 1:500 dilution of Intercalator-Ir (Fluidigm) in TBS for 2 min at RT. Slides were briefly washed with H_2_O and air-dried before IMC acquisition. Data were acquired on a Hyperion imaging system coupled to a Helios Mass Cytometer (Fludigm Hyperion Imaging System™), at a laser frequency of 200 Hz and laser power of 3 dB. For each recorded ROI, stacks of 16-bit single-channel TIFF files were exported from MCD binary files using MCD™ Viewer 1.0 (Fluidigm San Francisco, CA, USA).

## Results

**Figure 1:**
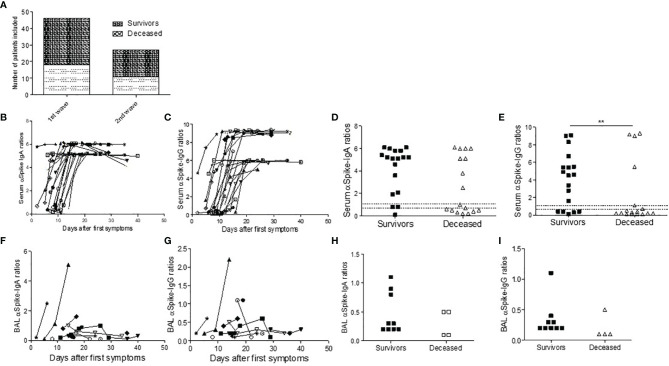
SARS-CoV-2 antibody response in ICU COVID-19 patients. Number of patients tested during the first and second wave **(A)**. **(B–I)** Antibodies against the S1 domain of the spike protein were measured in ELISA assay in first-wave survivors (closed symbols) or deceased (open symbols) patients at different time points from first COVID symptoms. **(B, C)** Serum IgA **(B)** and IgG responses **(C)** are depicted during ICU stay. **(D, E)** First time point IgA **(D)** and Ig IgG **(E)** titers in survivors and deceased patients. In BAL, the IgA **(F, H)** and IgG **(G, I)** antibody response was weak in deceased patients (open symbols) when compared to survivors (closed symbols). Chi-square test was used for serology comparison, while Mann–Whitney test was used for comparing BAL antibody levels.

We studied sequential blood samples from a total of 74 patients including 46 first and 28 second waves, respectively, with proven critical COVID 19 infection (median age 54 years, sex ratio 3.3, **Figure 1A**, **Tables 1**, **2**). We also analyzed BAL from 35 patients (11 deceased) at different time points during their ICU stay. The second-wave patients received steroids for the treatment of COVID-19 infection for a median 11 days.

We first studied the blood and BAL IgA and IgG spike-specific SARS COV2 serology (n=35). All first-wave patients were seroconverted within 15 days with high serum antibody levels, the IgG response being time-shifted when compared to the IgA response (**Figures 1B, C**). However, deceased patients displayed a trend for a lower IgA response (p=0.052, **Figure 1D**) and significantly lower SARS-CoV-2-specific IgG signal-to-cut-off ratios at the first serum sampling at ICU admission than survivors (p=0.004, **Figure 1E**) and a trend for a lower IgA response (p=0.052, **Figure 1D**). The BAL retrieved from first-wave deceased patients contained SARS-CoV-2-specific IgA and IgG (**Figures 1F–I**). We investigated whether these low levels of BAL antibody response were linked to immune complex lung deposition, but the BAL antibody titers did not increase after immune complex dissociation in the glycine buffer (**Supplementary Figure 1**). In contrast, we found no difference during the second wave between the survivors and the deceased patients (**Supplementary Figure 2**).

**Figure 2:**
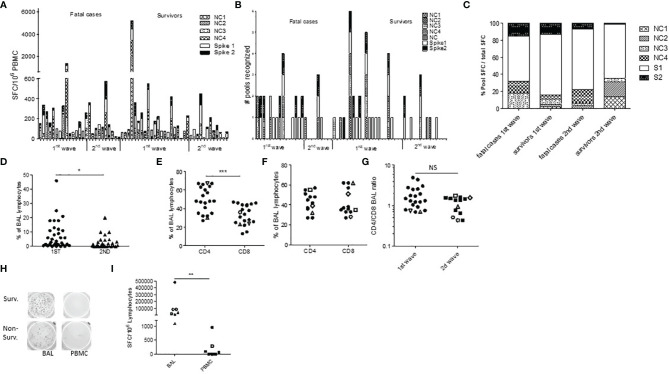
SARS-CoV-2-specific T cells trafficking to the lungs in severe COVID-19 infection. **(A, B)** PBMC ELISpot IFN-γ assays against spike protein (S, 2 peptide pools) and nucleocapsid (NC, 4 peptide pools) were assessed in 36 first-wave ICU patients at first time point and in 24 second-wave ICU patients **(A, B)**. Results are expressed as SFC units **(A)** or as a number of recognized peptide pools **(B)**. **(C)** Percentage of the total SFC response against SARS-CoV-2 peptide pool in each group. **(D)** Proportion of lymphocyte (Ly) on first BAL elements in first-wave patients (round symbols) and second-wave patients (triangles). **(E, F)** %CD3+CD4+ and CD3+CD8+ T cell in BAL lymphocytes in all BAL samples from first wave **(E)** and second wave **(F)**, and comparison of the CD4/CD8 BAL ratio during the first and second waves **(G)**. Open symbols are deceased patients. **(H, I)** Comparison of BAL lymphocytes and PBMC SARS-CoV-2 ELISpot IFN-γ assays in two representative patients **(H)** and in all patients **(I)**. *p<0.05 ***p<0.001; NS, not significant.

Then, peripheral blood SARS-CoV-2-specific cell-mediated responses were measured in 58 patients according to PBMC availability using an ELISpot-IFNγ assay and pools of 18 amino-acid long peptides covering the nucleocapsid (NC) and/or spike(S) antigens. We detected responses against the NC and S in 26/36 (72%) first-wave patients, including deceased patients, but only 8/22 (36%) in second-wave steroid-treated patients (**Figure 2A**). Individually, effector responses were mainly directed against the S1 spike protein and there was no difference between groups (first/second wave, deceased/survivors, **Figures 2B, C** and **Supplementary Figure 4**). Seven non-exposed donors were tested similarly and showed 2 NC responses (**Supplementary Table 2**).

To further describe the local pulmonary immune responses, we first phenotyped BAL cells and found a lower proportion of BAL lymphocytes during the second wave in steroid-treated patients (p=0.012, **Figure 2D**). There was also a trend for lower BAL lymphocyte percentages in non-survivors (p=0.191, **Supplementary Figure 3A**). The CD4/CD8 ratio among BAL lymphocytes showed a large predominance of CD4 over CD8 T cells during the first wave (p=0.0003, mean ratio 1.80, **Figure 2E** and **Supplementary Figure 3B**), while similar CD4 and CD8 BAL T-cell percentages were observed in steroid-treated patients during the second wave (median CD4 43%, mean ratio 1.14, non-significant with the first wave, **Figure 2F** and **Supplementary Figure 3B**). Accordingly, the CD4/CD8 ratio elevation seems more pronounced during the first wave (**Figure 2G**). Importantly, when available, those alveolar T cells could be analyzed in 7 cases for their specificities and showed high frequencies of SARS-CoV-2-specific T cells, both against the spike (median 56% of the BAL T- cell response) and the nucleocapsid protein (median 44% of the BAL T cell response) and both in survivors (n=4) and non-survivors (n=3) (**Figure 2H**). The frequencies of those specific T cells were more than 10-fold higher in BAL lymphocytes than in PBMCs (**Figure 2I**), even in steroid-treated patients.

**Figure 3:**
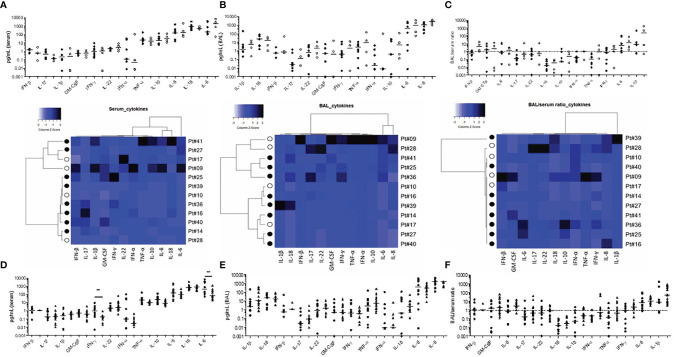
Serum and bronchoalveolar supernatant cytokine levels in severe COVID-19 infection. First-wave serum **(A)** and BAL **(B)** cytokine levels in survivors (n=8, black symbols), and deceased patients (n=4, open symbols). The BAL/serum ratio is shown in **(C)**. Heatmaps represent individual data of survivors and deceased patients and cytokine orders in whole **Figure 3** follow the hierarchical clustering of the corresponding heatmap. Cytokine serum **(D)**, BAL **(E)**, and BAL/serum ratio **(F)** levels for first- wave patients (rounded symbols) and second-wave patients (triangles).

We next assessed the serum (**Figure 3A**) and BAL (**Figure 3B**) cytokine levels and found similar profiles between 12 survivors and deceased first wave patients despite some BAL/serum ratios were either below (IL-18) or above 1 (IL-8, IL-1b, **Figure 3C**). Noteworthy, in the 15 second-wave steroid-treated patients studied, we found significantly lower IL-6 and IFN-γ serum levels when compared to the second-wave levels (p<0.007, **Figure 3D**), showing the anti-inflammatory and the peripheral modulation of the Th1 cell responses of steroids during severe COVID-19 infection. On the contrary, the BAL cytokine levels were similar in steroid-treated patients (**Figure 3E**), concordant with the persistence of local pulmonary inflammation under treatment (**Figure 3F**).

**Figure 4:**
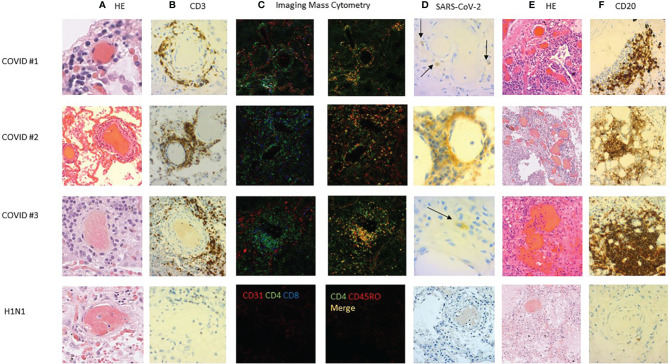
Lung histopathology of deceased COVID-19 cases. Lung sections of three COVID-19 cases were studied with hematoxylin–eosin staining (HES) and immunostaining. **(A)** HES staining showed sparse (case #1) or abundant (cases #2 and #3) lymphocytic vasculitis of middle-sized pulmonary vessels. **(B)** The lymphocytic infiltration was mainly CD3 positive **(C)** and also CD45RO+CD4+ positive in imaging mass cytometry **(C)**. **(D)** Immunostaining showed SARS-CoV-2-positive cells around middle-sized vessels (arrows). **(E)** Some vascular formations were surrounded **(F)** by CD20+ lymphocytes accumulating in what could be a tertiary lymphoid structure. This perivascular inflammation was not observed in an influenza H1N1 case (bottom panel).

To further characterize *in situ* the pulmonary immune environment during a fatal COVID-19 infection, we performed three autopsies of additional deceased non-steroid-treated patients (ages 58, 70, and 73). Two patients died in the ICU and one in standard hospitalization. The SARS-CoV-2 RT-PCR was positive on all three pulmonary samples, and diffuse alveolar damage lesions were observed and accompanied with vascular thrombosis in one patient. In all three cases, we observed a CD3+ T lymphocytic vascular inflammation with perivascular infiltrates, surrounding middle-sized vessels, some of them colocalizing with thrombosis (**Figures 4A, B**). Those cells were characterized as CD3+CD4+CD45RO+ memory T cells in imaging mass cytometry (**Figure 4C**). Noteworthy, these perivascular infiltrates were positive for SARS-CoV staining with the positivity of endothelial cells or perivascular cells (**Figure 4D**). Furthermore, several atypical aspects mimicking vascular malformations were observed in the lung parenchyma or in the subpleural tissue (**Figure 4E**). Those vascular structures were surrounded by the infiltrates of CD20+ cells (**Figure 4E**). We next retrieved archived lung sections from a case deceased from influenza H1N1 pulmonary infection in 2009. None of these perivascular lymphoid infiltrates were observed in this postmortem fragment (**Figure 4**).

## Discussion

Taken together, these results suggest a new immunopathological mechanism for pulmonary severe and fatal COVID-19 infection in an ICU. Indeed, we showed the alveolar recruitment of SARS-CoV-2-specific T cells against both the spike and the nucleocapsid. This may be due to the preferential replication of SARS-CoV-2 in alveolar pneumocytes and respiratory epithelial cells, as well as in endothelial cells as observed in lung fragments. This T-cell response was a Th1 response as assessed by the ELISpot-IFNg assay and might be predominantly of CD4 T-cell origin as suggested by the immune phenotyping. Also consistent with this hypothesis, the 18 amino-acid long peptides we used might stimulate better the CD4 T cells than the CD8 ones. The IFN-γ, IL-22, and IL-10 levels we found in BAL might also suggest the presence of some TfH and Tregs or Th2 CD4 T cells in the lungs. Altogether, these data suggest that despite severe COVID-19 pneumonia, SARS-CoV-2- specific CD4 T cells migrate to the lungs but remain detectable in the periphery. We also show that steroid treatment seems to damper systemic inflammation and specific CD4 T-cell responses, with a lower percentage of BAL lymphocytes during the second wave, even if an increase of non-lymphoid cells could not be excluded. We found no other explanations for the difference between the two waves with the same virus strain, the Wuhan original strain. The delay from first symptoms was longer during the second wave (p<0.001, **Tables 1**, **2**), probably because of slightly different recruitment (all but one ECMO patient during the second wave). However, and taken together, our results suggest that the pathogenic role of CD4 T lymphocytes could be evoked during severe COVID-19 because steroids classically damper the T-cell responses and are associated with a better outcome.

Accordingly, during fatal forms, the strong alveolar T-cell response we describe is indistinguishable from the survivors’ one, suggesting that a lack of specific response is not responsible for the fatality. In contrast, the 3 additional autopsy cases allowed us to describe for the first time a pulmonary vasculopathy with a memory CD3+CD4+CD45RO+ T-cell lymphocytosis. This was not described by others with the same technique (Rendeiro Nature 2021, Wang Front Microbiol 2020) with, however, the description of a macrophage lung infiltration and a lack of CD8 infiltration during COVID-19 infection. This particular pattern we observed may be linked to the systemic and local virus replication, as pulmonary endothelial cells being infected in higher rates, and might be favored by endothelial injury in the context of vascular comorbidities. This angiopathy may be linked to the vascular thrombosis observed in more severe forms of COVID-19 infection (22). Furthermore, this pulmonary memory CD4 response observed in fatal forms of the disease is reminiscent of a work by Bacher et al. showing that low-avidity SARS-CoV-2-specific CD4 T cells are detectable in periphery only during more severe forms of the disease. A low avidity profile was also observed for SARS-CoV-2-specific T cells in non-exposed donors, suggesting that pre-expanded CD4 T-cell responses could accumulate during COVID-19 in the context of inflammation and be inefficient to clear the virus (23). This is hypothesis is corroborated by the SARS-CoV-2- specific T cells we and others observed in non-exposed donors. We propose that those cells could accumulate in the lungs at both the alveolar and perivascular levels during more severe forms of the disease.

We also show herein local alveolar specific immunoglobulins and confirm the precociously detectable humoral SARS-CoV-2-specific systemic response (24). A lower IgG and IgA serum dosage was observed during the first wave in deceased patients, although we could exclude immune complex lung deposition. Such a lack of immune complex deposition differs from our previous findings in lethal cases of H1N1 pneumonia (16). These differences were not observed in steroid-treated patients, suggesting a beneficial effect of steroids. Interestingly, we show here that the local humoral response may be linked with sparse perivascular B-cell formations surrounding the T-cell infiltrates, which could correspond to the tertiary lymphoid structures reported in the lungs of animal models of influenza infection (25). Supporting also this concept is the predominance of CD4 T cells in BAL and the levels of IL-22, suggesting that some TfH cells might be at play. Hence, the local specific response to the virus seems to be organized appropriately during severe COVID-19 pneumonia.

There are limitations in such a study because we could not have access to pulmonary biopsies in surviving patients or in steroid-treated patients. One can suspect that the lung T-cell angiopathy occurs in surviving ICU forms and that the hallmark of fatal forms is rather a pulmonary memory CD4 T-cell infiltrate responding to the virus. We could not determine the exact site of viral replication in this study. However, the viral staining on endothelial cells and in the perivascular area in three deceased patients allows us to propose that the observed T-cell pulmonary angiopathy could be the consequence of an *in situ* migration of effector/memory T cells, thus corroborating the hypothesis of a preferential pulmonary vascular virus replication in this context. Whether the T-cell perivascular migration occurs precociously in the onset of the disease and whether comorbidities such as diabetes, hypertension, or age impact this phenomenon remain to be determined. Finally, the impact of steroids on the local T-cell response may be addressed in a future randomized study.

In summary, our study proposes a new immunopathological mechanism for fatal forms of COVID-19 infection. The demonstration of high frequencies of SARS-CoV-2-specific T cells at the alveolar level shows that despite the severity, an appropriate or even pathogenic T-cell trafficking to the lungs could be at play. The novel demonstration of a memory CD4 T-cell pulmonary angiopathy colocalizing with SARS-CoV-2 in a context of active immune response opens new insights for future therapeutic interventions.

## Data Availability Statement

The original contributions presented in the study are included in the article/**Supplementary Material**. Further inquiries can be directed to the corresponding author.

## Ethics Statement

The protocol was approved by our institution’s ethics committee (Immuno-COVID-REA, CER-Sorbonne Université, no. CER-SU-2020-31). The patients/participants provided their written informed consent to participate in this study.

## Author Contributions

AG, IP, DS, A-GM, GG, and C-EL designed the study. CS, AR, CN, IM, AG, KD, CP, and PH did the experiments. LA, OB, and PR did biobanking. AG, BC, PD, BA, A-GM, and C-EL compiled the data and discussed the results. IP, DS, and FC realized the autopsies and pathological examinations. JM, AB, GV, AL, and C-EL recruited ICU patients. AG and CEL wrote the paper. All authors contributed to the article and approved the submitted version.

## Conflict of Interest

The authors declare that the research was conducted in the absence of any commercial or financial relationships that could be construed as a potential conflict of interest.

## Publisher’s Note

All claims expressed in this article are solely those of the authors and do not necessarily represent those of their affiliated organizations, or those of the publisher, the editors and the reviewers. Any product that may be evaluated in this article, or claim that may be made by its manufacturer, is not guaranteed or endorsed by the publisher.

## Supplementary Material

Supplementary Figure 1 ELISA IgG and IgA SARS-CoV-2-specific antibody content of BAL before and after immune complex dissociation. This was performed in order to explore the possibility of pathogenic immune complex deposition in more severe cases of COVID-19 infection, as observed for severe influenza infection. Antibody levels were not higher after immune complex dissociation with buffer, in survivors (closed symbols) as in deceased patients (open symbols).

Supplementary Figure 2 SARS-CoV-2 antibody response in second wave intensive care unit COVID-19 patients. Antibodies against the S1 domain of the spike protein was measured in ELISA assay in second wave survivors (closed symbols) or deceased (open symbols) patients (n=35) at different time points from first COVID symptoms. Serum IgA **(A)** and IgG responses **(B)** are depicted during ICU stay. **(C, D)** First time point IgG **(C)** and IgA **(D)** titers in survivors and deceased patients. In broncho-alveolar lavage (BAL), the IgA **(E, G)** and IgG **(F, H)** antibody response was weak in deceased patients (open symbols) when compared to survivors (closed symbols).

Supplementary Figure 3 Lymphocyte percentages in BAL elements from severe COVID-19 patients. Survivors are closed symbols, non survivors are open symbols. First wave and second wave patients are represented **(A)**. BAL lymphocyte phenotyping in a representative patient from the first wave. T cell lymphocytes are analyzed on CD45+ cells of total BAL cells No B cells or NK cells were detected **(B)**.

Supplementary Figure 4 Repartition of the peripheral effector and effector-memory T cell responses to SARS-CoV-2 measured in ELISpot-IFNγ assay and tested against 18-mer peptides covering the nucleocapsid (NC) and spike (S) proteins, expressed as the percentage of the total response. NC peptides were divided in 4 pools and S peptides in 2 pools. NC peptides were tested in one superpool in one patient. Results are expressed as percentages of the magnitude (SFC/10^6^ PBMC) of the total SARS-CoV-2 cellular specific response. Patients groups are divided in first wave/second wave patients and in survivors/non survivors.

Supplementary Table 4 SARS-CoV-2-specific ELISpot assay in non-exposed donors (SFC/106^^^6PBMC).

Supplementary Table 5 Antibodies used for mass imaging cytometry.
