# Supplementary figures and images for: Memory CD4+ T-Cell Lymphocytic Angiopathy in Fatal Forms of COVID-19 Pulmonary Infection
Source: Front Immunol. 2022 Apr 22;13:844727. doi: 10.3389/fimmu.2022.844727 (PMC9074842; doi:10.3389/fimmu.2022.844727)

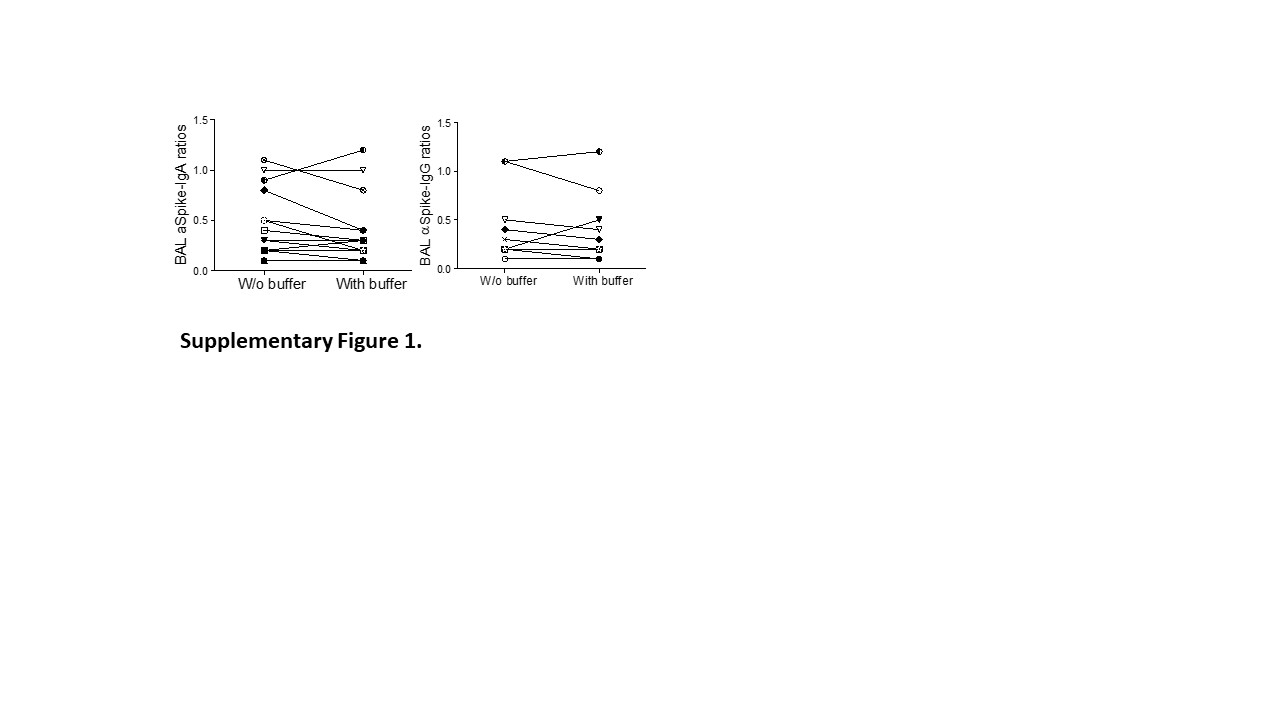

Supplement: Supplementary Figure 1 — ELISA IgG and IgA SARS-CoV-2-specific antibody content of BAL before and after immune complex dissociation. This was performed in order to explore the possibility of pathogenic immune complex deposition in more severe cases of COVID-19 infection, as observed for severe influenza infection. Antibody levels were not higher after immune complex dissociation with buffer, in survivors (closed symbols) as in deceased patients (open symbols). [file Image_1.jpeg]

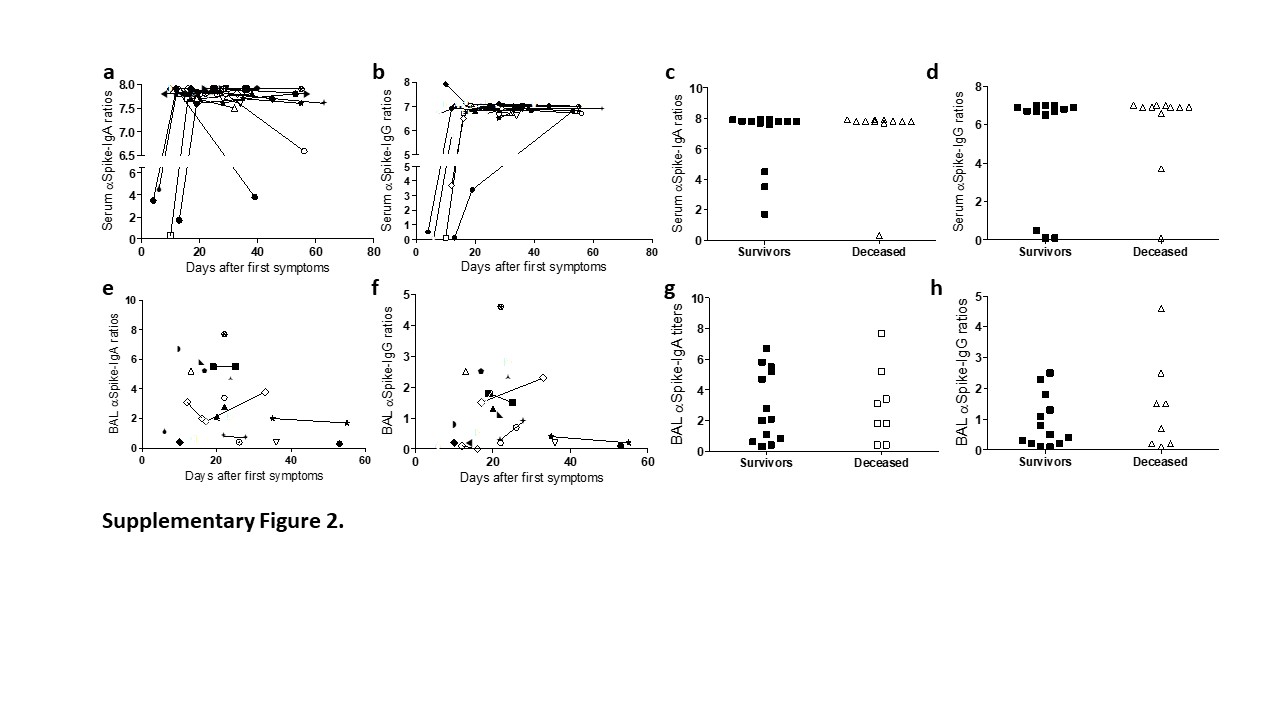

Supplement: Supplementary Figure 2 — SARS-CoV-2 antibody response in second wave intensive care unit COVID-19 patients. Antibodies against the S1 domain of the spike protein was measured in ELISA assay in second wave survivors (closed symbols) or deceased (open symbols) patients (n=35) at different time points from first COVID symptoms. Serum IgA (A) and IgG responses (B) are depicted during ICU stay. (C, D) First time point IgG (C) and IgA (D) titers in survivors and deceased patients. In broncho-alveolar lavage (BAL), the IgA (E, G) and IgG (F, H) antibody response was weak in deceased patients (open symbols) when compared to survivors (closed symbols). [file Image_2.jpeg]

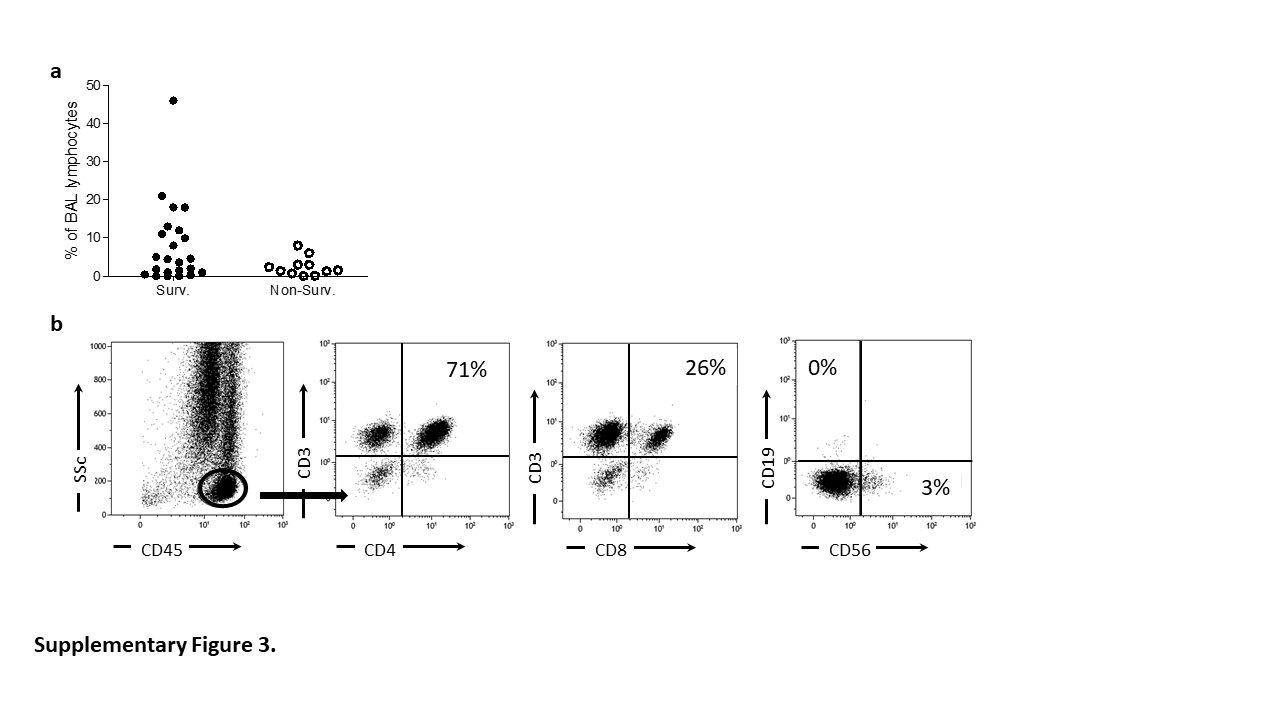

Supplement: Supplementary Figure 3 — Lymphocyte percentages in BAL elements from severe COVID-19 patients. Survivors are closed symbols, non survivors are open symbols. First wave and second wave patients are represented (A). BAL lymphocyte phenotyping in a representative patient from the first wave. T cell lymphocytes are analyzed on CD45+ cells of total BAL cells No B cells or NK cells were detected (B). [file Image_3.jpeg]

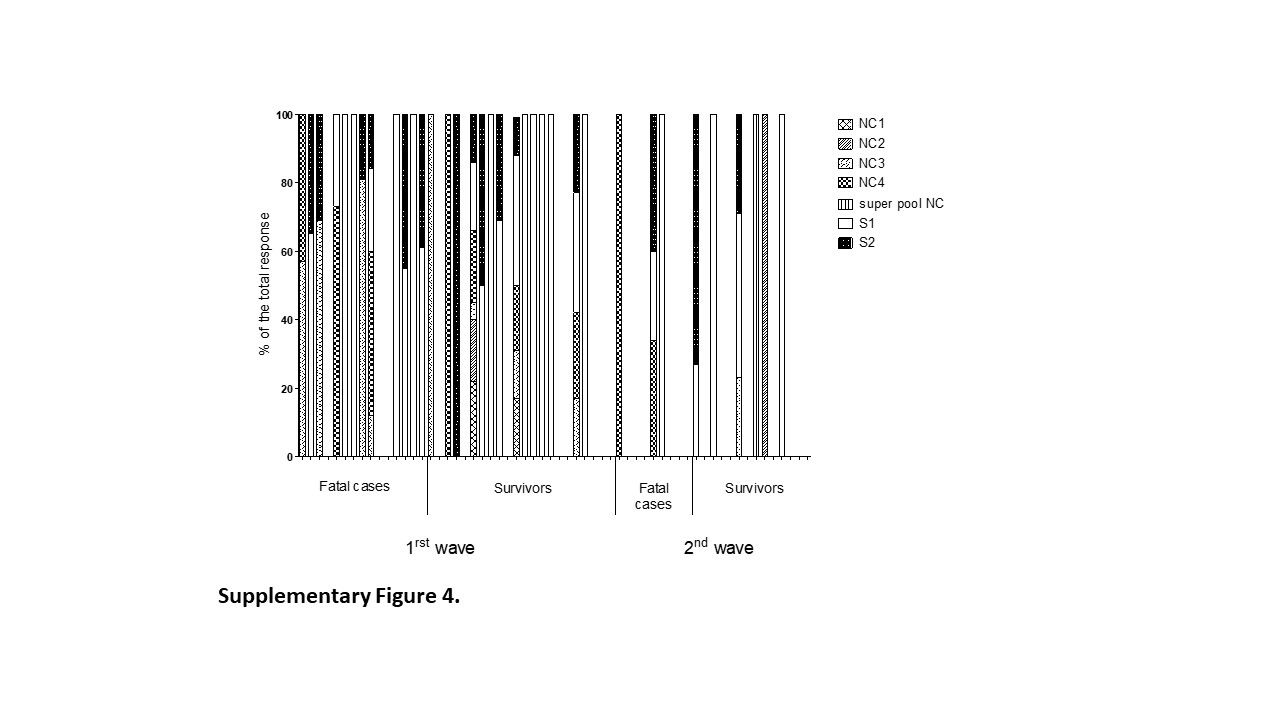

Supplement: Supplementary Figure 4 — Repartition of the peripheral effector and effector-memory T cell responses to SARS-CoV-2 measured in ELISpot-IFNγ assay and tested against 18-mer peptides covering the nucleocapsid (NC) and spike (S) proteins, expressed as the percentage of the total response. NC peptides were divided in 4 pools and S peptides in 2 pools. NC peptides were tested in one superpool in one patient. Results are expressed as percentages of the magnitude (SFC/106 PBMC) of the total SARS-CoV-2 cellular specific response. Patients groups are divided in first wave/second wave patients and in survivors/non survivors. [file Image_4.jpeg]
